# Supplementary material for: Elevated Plasma Bioactive Adrenomedullin and Mortality in Cardiogenic Shock: Results from the OptimaCC Trial
Source: J Clin Med. 2021 Sep 29;10(19):4512. doi: 10.3390/jcm10194512 (PMC8509471; doi:10.3390/jcm10194512)
Supplement: Supplementary file 1 [file jcm-10-04512-s001.zip › jcm-1359502-supplementary.pdf]

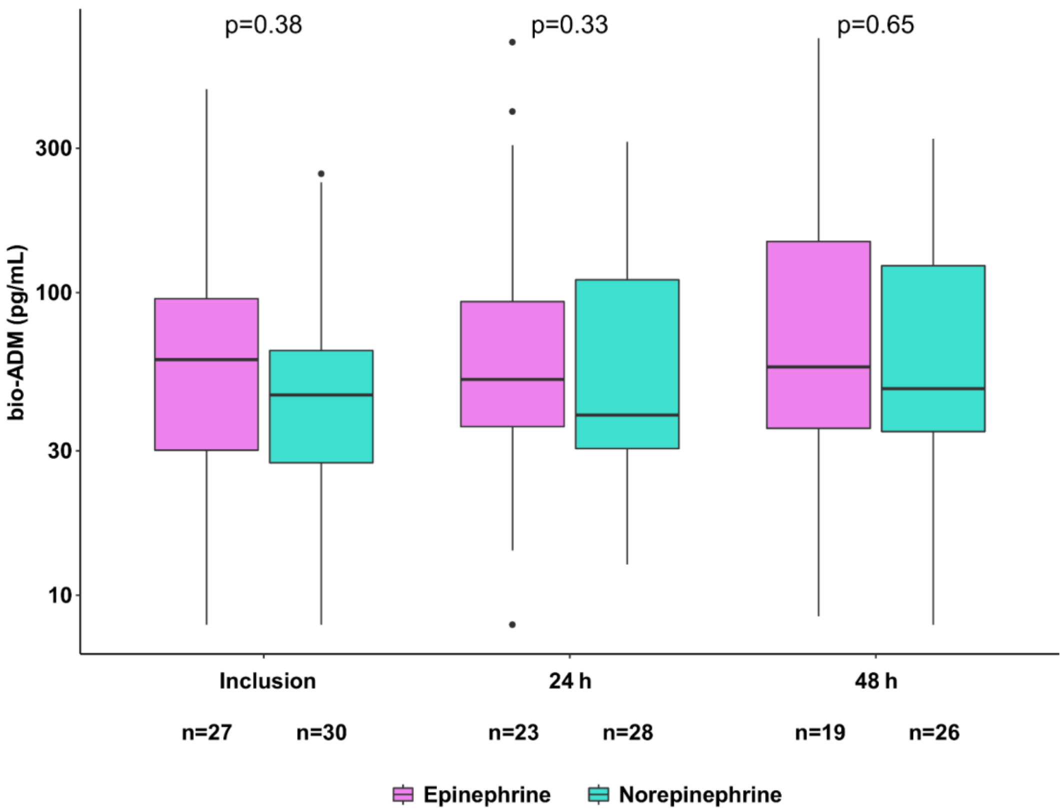

Figure S1. Comparison of time-course of bio-ADM, from inclusion to 48 h, between epinephrine group and norepinephrine group. A base-10 log scale is used for the y-axis. Comparisons at each time point were performed using the Kruskal-Wallis test comparing bio-ADM values in epinephrine group versus bio-ADM in norepinephrine group. bio-ADM, bioactive adrenomedullin.

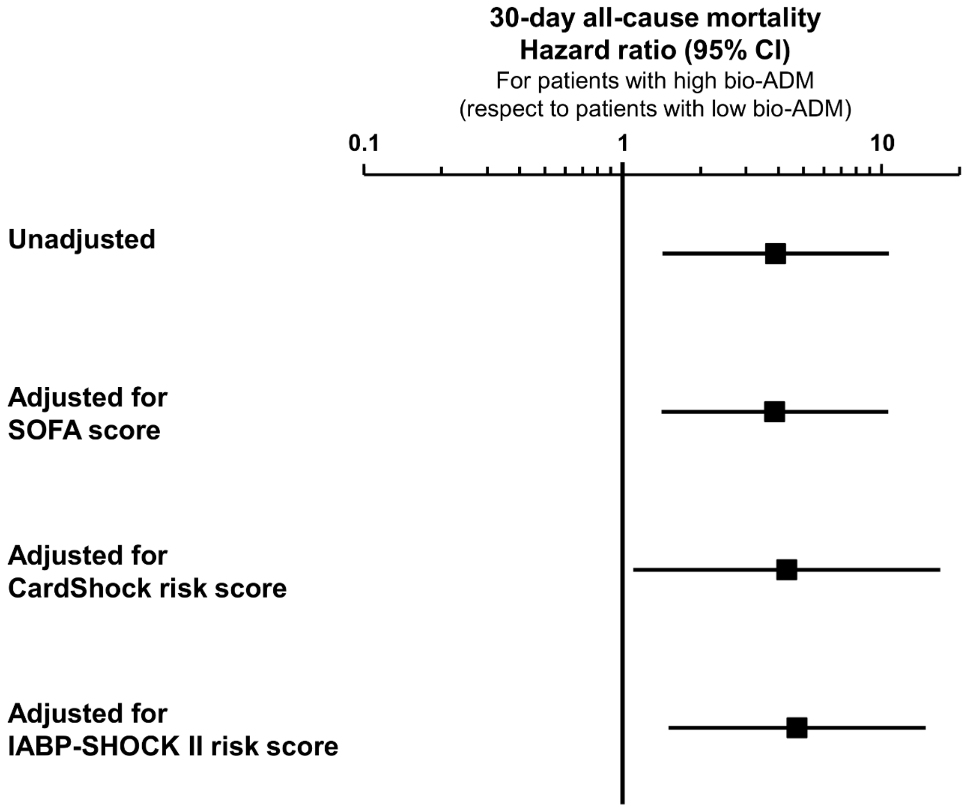

Figure S2. Unadjusted and adjusted ratios for high bio-ADM value (≥ 53.8 pg/mL) at inclusion compared with low bio-ADM (<53.8 pg/mL) for outcome: 30-day all-cause mortality. Dichotomization of patients was based on bio-ADM value 53.8 mg/mL, which was the median value of bio-ADM at inclusion (interquartile range 28.5–85.2 pg/mL). bio-ADM, bioactive adrenomedullin; CI, confidence interval; HR, hazard ratio; SOFA score, sequential organ failure assessment score.

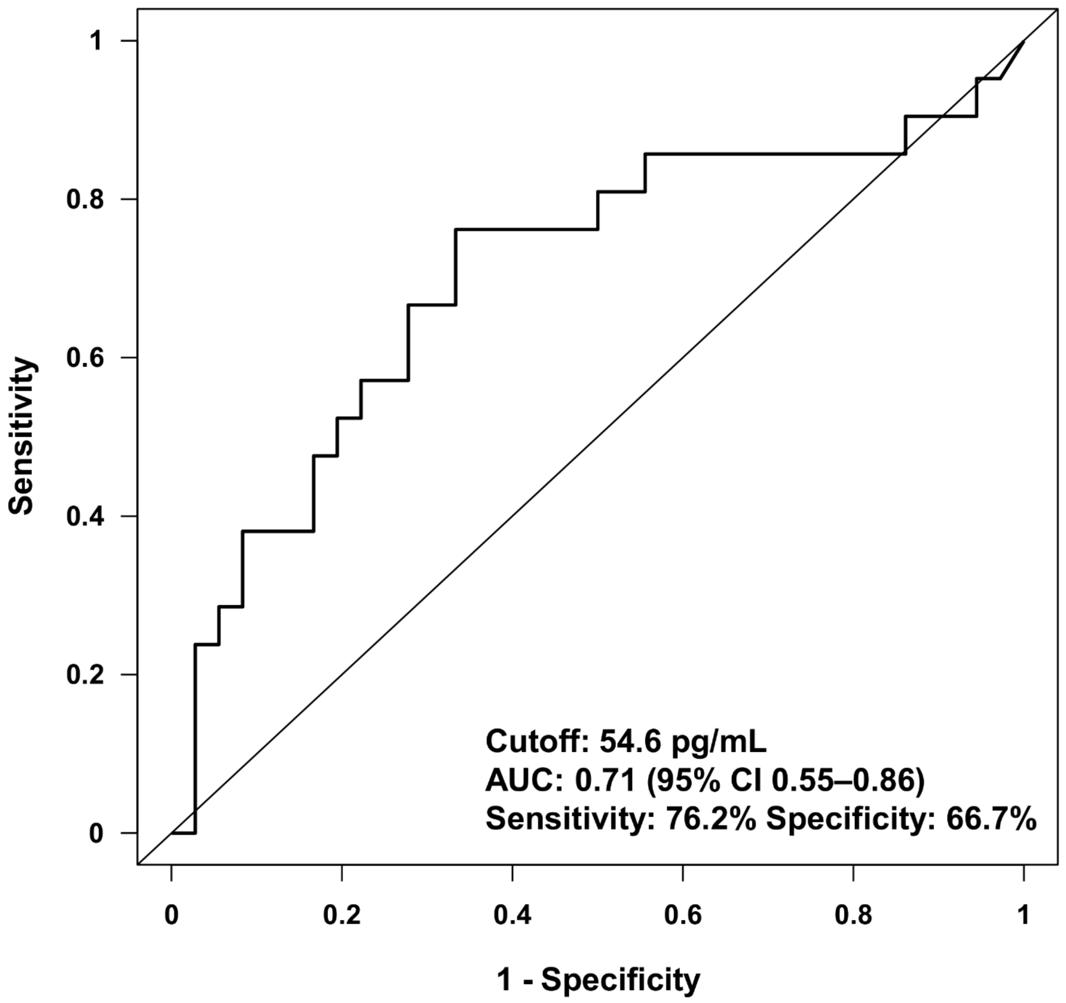

Figure S3. Receiver operating characteristic curve for determining the cut-off point of the bio-ADM for 30-day all-cause mortality. AUC, area under the receiver operating characteristic curve; bio-ADM, bioactive adrenomedullin; CI, confidence interval.

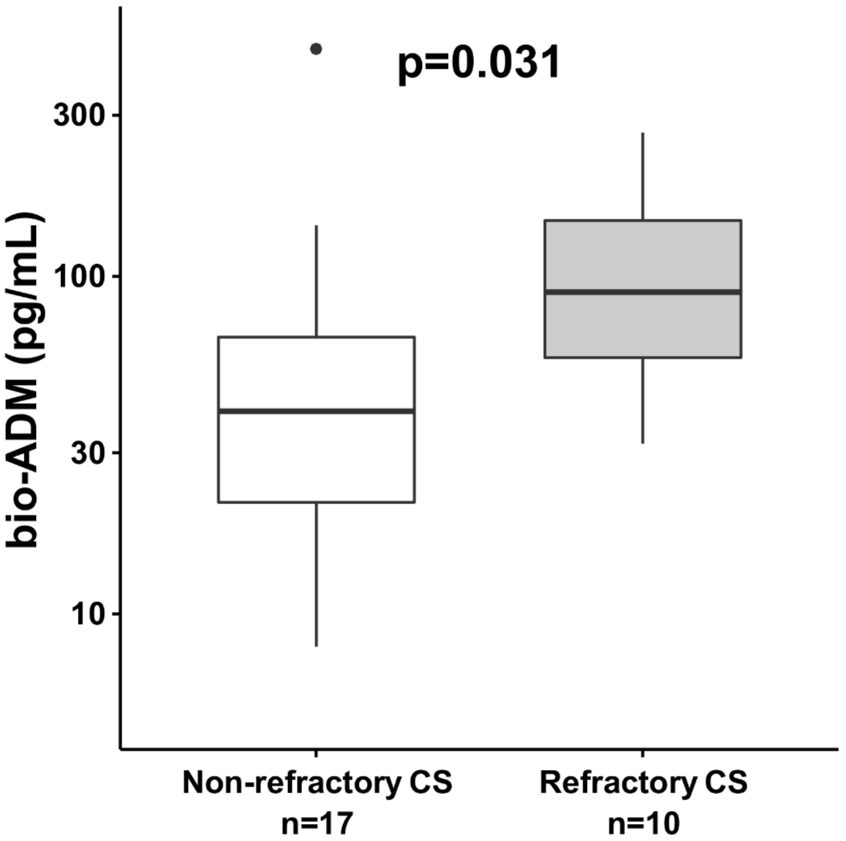

Figure S4. The value of bio-ADM at inclusion between patients with and without refractory cardiogenic shock in epinephrine group. bio-ADM, bioactive adrenomedullin; CS, cardiogenic shock.

Table S1. Comparison of characteristics of cardiogenic shock patients between high bio-ADM and low bio-ADM groups at inclusion.

Variables	High bio-ADM *n* = 29	Low bio-ADM *n* = 28	*p*-value
Demographics			
Age (years)	75 [67–81]	56 [52–67]	<0.001
Female gender	12 (41)	7 (25)	0.26
Medical history			
Hypertension	10 (34)	4 (14)	0.12
Diabetes	5 (17)	1 (4)	0.19
Stroke	2 (7)	2 (7)	1.00
Myocardial infarction	3 (10)	1 (4)	0.61
Severity scores			
SOFA score	10 [8–12]	9 [8–11]	0.61
CardShock risk score	5 [4–7]	4 [3–4]	0.003
IABP-SHOCK risk score	2 [1–4]	1 [0–3]	0.070
Clinical presentation at inclusion			
Body mass index (kg/m2)	26.6 [23.7–27.6]	24.0 [20.8–26.8]	0.068
Heart rate (bpm)	96 [84–116]	97 [80–106]	0.97
Systolic arterial pressure (mmHg)	110 [97–116]	98 [94–118]	0.52
Diastolic arterial pressure (mmHg)	55 [46–61]	60 [55–69]	0.045
Mean arterial pressure (mmHg)	70 [63–80]	72 [69–89]	0.12
SvO_2 (%)	69 [57–76]	73 [66–80]	0.084

Mechanical ventilation	20 (83)	26 (96)	0.17
LVEF (%)	32 [24–45]	37 [29–40]	0.36
Laboratory findings at inclusion			
eGFR (mL/min/1.73m^2)	39.8 [31.1–49.0]	69.5 [53.3–90.5]	<0.001
AST (UI/L)	456 [143–1076]	471 [251–704]	0.99
ALT (UI/L)	144 [68–322]	149 [80–210]	0.94
NT-proBNP (pg/mL)	7543 [4427–20766]	702 [375–1782]	<0.001
hs-TnT (pg/mL)	8.7 [2.3–33.8]	6.9 [2.9–17.9]	0.54
Lactate (mmol/L)	4.6 [2.9–5.8]	2.9 [1.9–5.4]	0.08
Adverse events in ICU			
Refractory cardiogenic shock	10 (34)	2 (7)	0.021
Arrhythmias	13 (45)	8 (29)	0.27
MCS implantation	2 (7)	2 (7)	1.00

Data are presented as median [interquartile range] or *n* (%). High bio-ADM corresponds to bio-ADM ≥ 53.8 pg/mL (median) and low bio-ADM corresponds to < 53.8 pg/mL. Arrhythmias indicates ventricular tachycardia, ventricular fibrillation, or atrial fibrillation. AST, aspartate transaminase; ALT, alanine transaminase; eGFR, estimated glomerular filtration rate; hs-TnT, high-sensitive troponin T; IABP-SHOCK II, intra-aortic balloon pump in cardiogenic shock II; IQR, interquartile range; LVEF, left ventricular ejection fraction; MCS, mechanical circulatory support; NT-proBNP, N-terminal pro-B-type natriuretic peptide; SOFA, sequential organ failure assessment; SvO2, mixed venous oxygen saturation.

Table S2. ROC curve analysis.

Model	AUC	95% CI (AUC)	Sensitivity (%)	Specificity (%)	*p*-value
bio-ADM	0.71	0.55–0.86	76.2	66.7	Reference
bio-ADM + NT-proBNP	0.73	0.58–0.87	66.7	74.3	0.69
bio-ADM + eGFR	0.72	0.57–0.86	89.5	51.5	0.96
bio-ADM + lactate	0.73	0.58–0.88	66.7	75.0	0.81

AUC, area under the curve; bio-ADM, bioactive adrenomedullin; CI, confidence interval; eGFR, estimated glomerular filtration rate; NT-proBNP, N-terminal pro-B-type natriuretic peptide; ROC, receiver operating characteristic.
